# Clavicle fracture at the suture hole after acromioclavicular joint reconstruction using a suture-button: a case report

**DOI:** 10.1186/s12891-019-2720-z

**Published:** 2019-07-18

**Authors:** Doji Inoue, Ryogo Furuhata, Kazuya Kaneda, Yoshihiro Ritsuno, Aki Kono, Yasuhiro Kiyota, Hideo Morioka, Hiroshi Arino

**Affiliations:** Department of Orthopaedic Surgery, National Hospital Organization Tokyo Medical Center, 2-5-1, Higashigaoka, Meguro-ku, Tokyo, 152-8902 Japan

**Keywords:** Suture-button, Suture-button fixation, Clavicle fracture, Acromioclavicular reconstruction, Suture-button complication

## Abstract

**Background:**

Intraosseous suture-button devices have been used for acromioclavicular joint reconstruction due to its relative simplicity compared with other procedures. However, the complications of acromioclavicular joint reconstruction using a suture-button are not fully understood. Here, we describe a case of a clavicle fracture at the suture hole following acromioclavicular joint reconstruction using a suture-button and hook plate.

**Case presentation:**

A 28-year-old man presented at our hospital after a fall from his bicycle. The patient had a history of acromioclavicular joint reconstruction with a suture-button and a hook plate for right acromioclavicular joint dislocation, seven months ago at another hospital. The hook plate had been removed four months ago, while X-ray radiography before removal had shown the widening of a suture hole. In the current fall from the bicycle, X-ray radiography revealed a clavicle fracture through the previous drill hole for suture-button. We removed the suture-button and performed an open reduction and internal fixation for the clavicle fracture.

**Conclusion:**

The present case indicated that a clavicle fracture at the suture hole, although rare, is one of the complications after an acromioclavicular joint reconstruction using a suture-button. This case suggested that drilling to the necessary minimum when making suture holes and paying attention to the widening of suture holes are important to prevent a postoperative clavicle fracture.

## Background

Intraosseous suture-button devices such as ZipTight™ (Zimmer Biomet, Warsaw, IN), TightRope® (Arthrex, Naples, FL), and Mini TightRope® (Arthrex, Naples, FL) have been widely used in tibiofibular fixation of ankle fractures, acromioclavicular joint (ACJ) reconstructions of acromioclavicular dislocations, corrective fixations for hallux valgus, and suspensionplasty in carpometacarpal osteoarthritis [1]. While favorable short-term outcomes were reported in ACJ reconstruction using a suture-button [2–4], complications such as a loss of reduction, pulling-out of the end button, clavicle fractures, and coracoid process fractures were also reported [2, 4–12].

In recent years, in order to resolve the pulling-out of the end button, procedures using a plate in addition to a suture-button have also been reported [13]. However, long-term outcomes and complications in cases using a plate concurrently, remain unknown.

Here, we describe a case of clavicle fracture originating from the suture-button entry-point, following acromioclavicular reconstruction using a hook plate and a suture-button.

Written informed consent was obtained from the patient for publication of this case report and any accompanying images.

## Case presentation

**Fig. 1:**
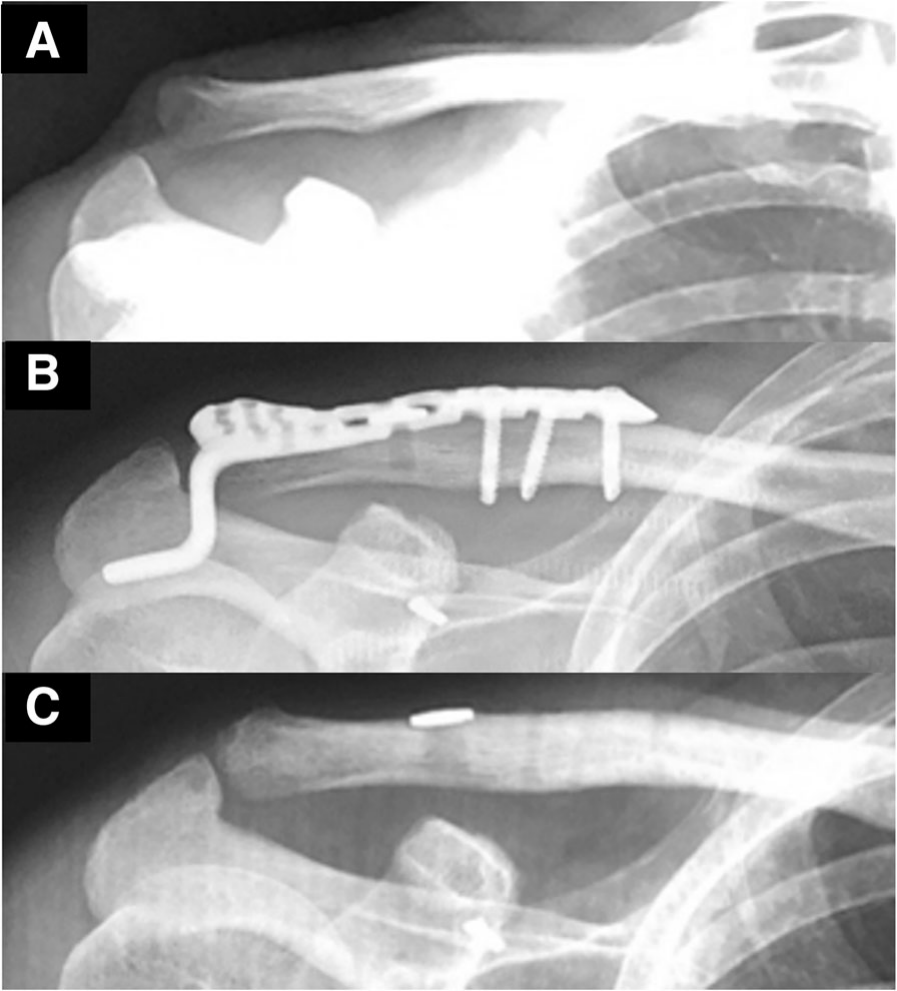
First injury and its treatment. X-ray radiography image at the initial injury demonstrated right acromioclavicular joint (ACJ) dislocation (**a**), and the patient underwent ACJ reconstruction using a suture-button and a hook plate seven months ago (**b**). The hook plate was removed four months ago (**c**)

A 28-year-old man presented at our hospital after a fall from his bicycle. He complained of right shoulder pain and the inability to move his right arm. The patient had a history of right ACJ dislocation because of a fall from his bicycle seven months earlier (Fig. 1a). He had undergone a right ACJ reconstruction with a suture-button (Zimmer Biomet, Warsaw, IN) and a hook plate (HOYA Technosurgical, Shinjuku, Tokyo, Japan) at another hospital (Fig. 1b). The suture-button passed from the clavicle to the coracoid via a 4.5 mm drill hole. After ACJ reconstruction, the hook plate was removed four months ago while X-ray radiography image before the removal suggested widening of the suture hole (Fig. 1c).

**Fig. 2:**
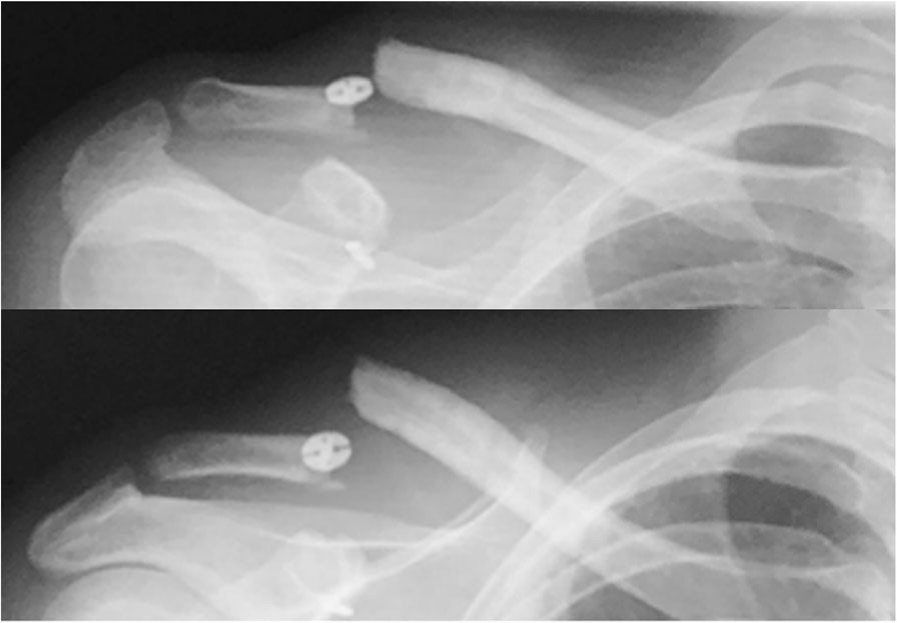
X-ray radiography images at the second injury revealed a clavicle fracture at the suture hole

At the time of the present visit, a deformity of the right distal clavicle and loss of range of the right shoulder motion were observed. There were no findings suggestive of nerve or vascular injury. X-ray radiography images revealed a right clavicle fracture at the suture hole (Fig. 2). Due to significant dislocation of the clavicle, removal of a suture-button, open reduction and internal fixation were scheduled.

**Fig. 3:**
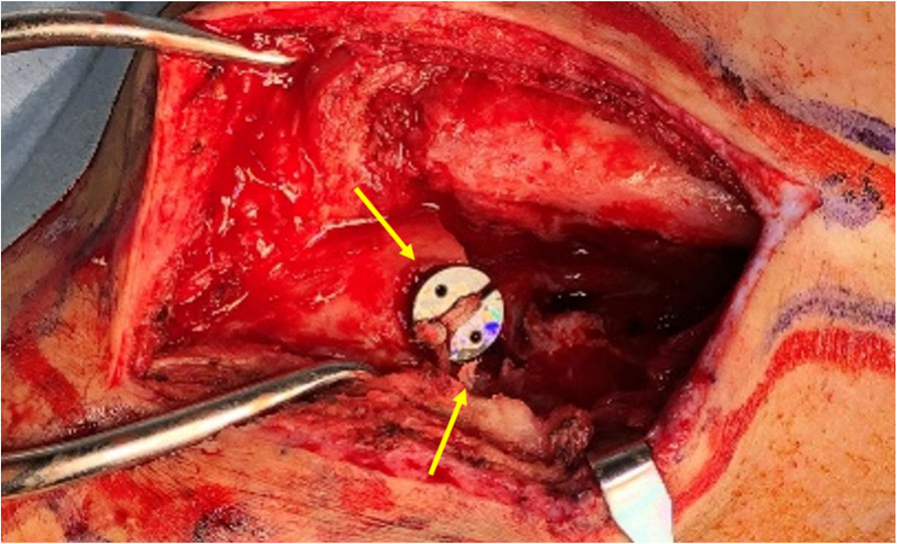
Intraoperative finding of multiple entry-point drilling holes near the suture hole

**Fig. 4:**
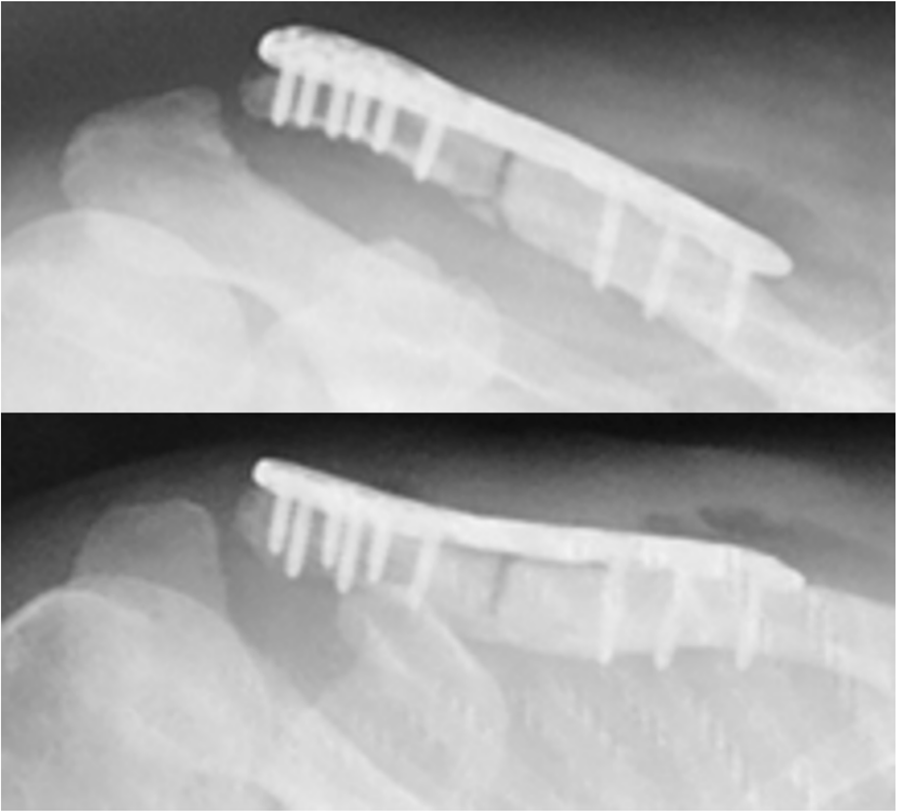
Intraoperative x-ray radiography images from the present surgery. X-ray radiography images after open reduction and internal fixation of the right clavicle revealed that the clavicle fracture was reduced and fixed by a plate

Intraoperative findings demonstrated a fracture through the suture hole and multiple drilling holes near the suture hole (Fig. 3). After the end-button of the coracoid process was detected and gripped with forceps, the suture was cut and the suture-button removed. Thereafter, open reduction was performed, and internal fixation was completed with a plate (Stryker, Kalamazoo, MI) (Fig. 4).

## Discussion and conclusions

The present case indicated two clinical issues. First, the frequency of a clavicle fracture originating from the drill hole is rare; however, it is one of the complications to be aware of, after ACJ reconstruction using a suture-button. There have been several cases of second metatarsal fracture in the correction of the hallux valgus [1, 14] and index metacarpal fracture in the carpometacarpal joint [15–17] reported as fractures near the suture-button. In particular, favorable reduction of the hallux valgus angle was observed in the correction of the hallux valgus using a suture-button, however, a second metatarsal fracture was observed in 32% of the cases postoperatively [1]. Meanwhile, there were two cases of coracoid process fractures and three cases of clavicle fractures reported as a fracture at the suture-button hole after ACJ reconstruction using a suture-button (Table 1). The frequency of the coracoid process fractures is 1.7% [10] and 1.9–5.6% for clavicle fractures [9, 12]. This is the first case of a clavicle fracture after using a suture-button and a hook plate. The present case occurred after a second trauma, seven months after the first surgery, when ligament repair could be completed. Previous clavicle fracture cases also occurred upon trauma 4–8 months postoperatively, suggesting that a clavicle fracture originating from the suture hole is a complication that can occur later, even after the coracoclavicular ligament has been repaired. In addition, all cases with clavicle fractures require open reduction and internal fixation using a plate. Therefore, a clavicle fracture at the entry-point of the suture-button is one of the notable complications that requires a reoperation.

**Table 1:** Previous reports’ summary of clavicular or coracoid fractures after acromioclavicular reconstruction with a suture-button

Author	Age (yr)/sex	Cause of fracture	Type of fracture	Postoperative time to fracture	Treatment
Ball et al. (2007) [6]	38/male	Fall from bicycle	Clavicle	8 months	Plate fixation
Bindra et al. (2011) [7]	32/male	Weightlifting	Coracoid	2 months	Conservative
Kany et al. (2012) [9]	N.D.	N.D.	Clavicle	N.D.	Plate Fixation
Martetschläger et al. (2013) [10]	37/male	Skiing injury	Coracoid	3 days	Conservative
Dunn et al. (2015) [11]	42/male	Fall	Clavicle	4 months	Plate Fixation
Current case	28/male	Fall from bicycle	Clavicle	7 months	Plate Fixation

*N.D.* not described

Second, in order to prevent clavicle fractures at the suture hole, it is essential to avoid excessive drilling of the suture hole during ACJ reconstruction and pay attention to the widening of suture holes at radiography images. Over-tensioning of the sutures [14, 16], osteolysis from a soft tissue reaction [15], technical factors due to the operator such as multiple entry-point drilling or suture holes that are not located in the center of the bone [1, 9], and poor adherence of the patients [7] are considered to be the mechanisms that can cause fractures near the suture hole. In the present case, multiple drilling holes were observed near the suture-button entry-point during the present surgery. According to previous reports investigating the effects of drilling and screw holes, bones are weakened by such holes when exposed to torsional and bending forces [18, 19]. Hence, multiple drilling near the suture hole may have caused a clavicle fracture in this case, indicating that drilling for a suture-button only to the necessary minimum is important. In addition, X-ray radiography images taken before the occurrence of the current clavicle fracture revealed widening of the suture hole. The tunnel widening is thought to be caused by multiple drilling attempts [13] or osteolysis from a soft tissue reaction [15] that in turn could have predisposed to a postoperative clavicle fracture in this case. Since the presence of non-absorbable sutures in the bone holes after operation can increase the risk of clavicle fractures, Ball et al. suggested that the suture-button should be removed 8 to 12 weeks after surgery, when the coracoclavicular ligament could be repaired [6]. However, considering that a loss of reduction may also occur following the removal of the suture-button, further research and investigation will be needed to address these issues. When inevitable multiple drilling was performed during the operation or widening of the suture hole was confirmed early after the operation as in the present case, a more cautious rehabilitation approach may be an option to adopt for 8–12 weeks after surgery, during which the coracoclavicular ligament can be repaired.

In summary, this case provides important information on the complication of ACJ reconstruction using a suture-button. Drilling to the necessary minimum when making suture holes and paying attention to the widening of suture holes are essential steps to prevent postoperative clavicle fractures near the suture-button in ACJ reconstruction.

## Data Availability

Date that support the findings of this study are available from the corresponding author on reasonable request.

## Abbreviation

ACJ: Acromioclavicular joint
